# Locomotion with Pedestrian Aware from Perception Sensor by Pavement Sweeping Reconfigurable Robot

**DOI:** 10.3390/s21051745

**Published:** 2021-03-03

**Authors:** Lim Yi, Anh Vu Le, Balakrishnan Ramalingam, Abdullah Aamir Hayat, Mohan Rajesh Elara, Tran Hoang Quang Minh, Braulio Félix Gómez, Lum Kai Wen

**Affiliations:** 1 ROAR Lab, Engineering Product Development, Singapore University of Technology and Design, Singapore 487372, Singapore; yi_lim@mymail.sutd.edu.sg (L.Y.); leanhvu@tdtu.edu.vn (A.V.L.); balakrishnan@sutd.edu.sg (B.R.); abdullahaamir@sutd.edu.sg (A.A.H.); rajeshelara@sutd.edu.sg (M.R.E.); braulio@lionsbot.com (B.F.G.); kaiwen_lum@mymail.sutd.edu.sg (L.K.W.); 2 Optoelectronics Research Group, Faculty of Electrical and Electronics Engineering, Ton Duc Thang University, Ho Chi Minh City 700000, Vietnam

**Keywords:** reconfigurable robot, sensors fusion, pavement cleaning, reconfiguration mechanism design, semantic segmentation deep neural network, feedback control

## Abstract

Regular washing of public pavements is necessary to ensure that the public environment is sanitary for social activities. This is a challenge for autonomous cleaning robots, as they must adapt to the environment with varying pavement widths while avoiding pedestrians. A self-reconfigurable pavement sweeping robot, named Panthera, has the mechanisms to perform reconfiguration in width to enable smooth cleaning operations, and it changes its behavior based on environment dynamics of moving pedestrians and changing pavement widths. Reconfiguration in the robot’s width is possible, due to the scissor mechanism at the core of the robot’s body, which is driven by a lead screw motor. Panthera will perform locomotion and reconfiguration based on perception sensors feedback control proposed while using an Red Green Blue-D (RGB-D) camera. The proposed control scheme involves publishing robot kinematic parameters for reconfiguration during locomotion. Experiments were conducted in outdoor pavements to demonstrate the autonomous reconfiguration during locomotion to avoid pedestrians while complying with varying pavements widths in a real-world scenario.

## 1. Introduction

The rise of housing and transportation facilities and infrastructure development in Singapore will create more new communities built, which means more pavement infrastructure. A newer initiative to promote walking and cycling as a mode for transportation in newer estates [1], as well as mature estates [2], will mean that there will be a growth of pavement in Singapore. In the article [3], the Land Transport Authority (LTA) of Singapore indicates that Singapore will continue to add to the existing 200 km network of sheltered walkways and with the continuous development of the Housing Development Board (HDB) community projects, the number is expected to grow. This requires a lot of workforce and cleaning equipment to continue keeping public pavements clean and safe for usage.

### 1.1. Precedence

In the fourth industrial revolution, automation has taken over many industries. Activities or tasks that are easily repeated can now be replaced with machines or software. Automation helps to drive efficiency, and this includes the cleaning industry. Cleaning is a repeated task and it is necessary for hygiene and can be automated. Especially in the current Covid 19 pandemic, there is a further push to automate cleaning in order to reduce the number of people exposed to the risk of the virus; hence, a lot of research and development has gone into pushing automation for cleaning activities. Most cleanings in parks are currently conducted using manual cleaning methods, where cleaners will drive around parks and pavements to sweep the floors. Park pavements usually vary between 60 cm to 300 cm in width, and the cleaners have to maneuver the cleaning vehicle in a winding manner in order to complete cleaning the pavement. Besides, cleaners have to stop now and then wait for pedestrians to leave the pavements to provide sufficient space for cleaning vehicles to perform cleaning operations. Research and development have pushed the frontiers of technology and, currently, there are semi-autonomous and autonomous cleaning robots that are capable of moving around an area to perform the cleaning. These robots operate in a pre-determined area, requiring a one-time setup to ensure that the robot can operate safely. Moreover, the length, width, and height of these robots are fixed. Thus, the robots cannot negotiate situations where the robot is cleaning in the pavement but blocked by an obstacle or pedestrian. They can only operate in a fixed minimum operating space and are unable to meet the needs of a robot to operate in dynamically changing pavement widths with obstacles.

A few commercial robots perform sweeping public pavements to tackle the increasing need for cleaning. However, these robots cannot negotiate various pavement widths and pedestrian density, causing problems where the robot blocks the pavement or the robot’s operation is limited, due to its fixed shape. Typical scenarios that are faced by cleaning robots are shown below.

**Figure 1:**
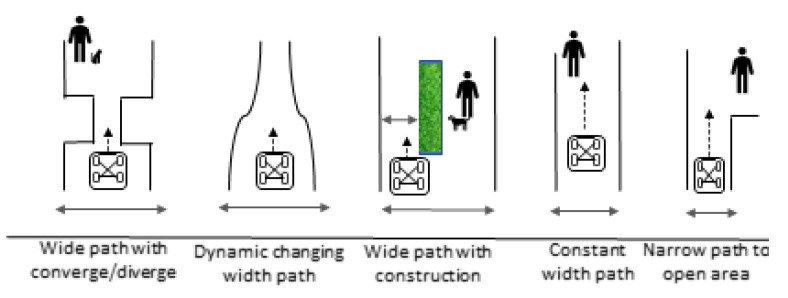
Common scenarios encountered by pavement sweeping vehicles.

As seen in Figure 1, the common scenarios faced by pavement cleaning robots are: 1. Large width pavement with pedestrians. 2. Width of the path that converges and diverges. 3. Small width pavement because of obstruction. 4. Straight small width pavement. 5. Small width pavement, which leads to a bigger space. 6. Small width pavement requires a small turning radius. 7. Pavement with moving obstructions such as pedestrians and pets.

Existing robots move in a zig-zag motion to cover the pavements’ area in order to clean these common pavement types. For pavements with obstructions or a pavement width that is too small, the robot will be unable to maneuver and perform cleaning operations. Fixed-sized robots cannot overcome typical scenarios that are faced by pavement cleaning robots where there is not enough space for the robot to move through. Some of these commercial robots, MN-E800W [4], QS3008 [5], and CN 201 [6], are studied and shown in the Table 1.

Commercial cleaning robots are mainly used for cleaning operations in Singapore. These commercial robots suffer from pain points due to the robot’s fixed dimension, where the robots are unable to perform a maneuver into a narrower pavement or obstructed pavement. Cleaning operators might need to stop occasionally when pedestrians are walking past them, and the cleaning robot does not have enough space to move without compromising pedestrians’ safety. Other times, there are static obstacles on the pavements where the robot does not have enough space to pass through. The cleaners will need to get off the cleaning robot and then manually move the obstacles or wait for the obstacle to move. Currently, robot autonomy in non-pre-determined surroundings is unstructured, and a lot of research and development have been invested in enabling robots to navigate smoothly.

### 1.2. Self-Reconfigurable Robots

Robots that have the ability to change their dimensions to tackle pain points are called self-reconfigurable robots. The purposes of shape reconfiguration varies, such as to perform another motion, such as climbing [7], rolling [8], floor cleaning [9], and floating [10]. One example is Scorpio [8]. Scorpio is able to perform locomotion on floors and walls, and it can transit between the two locomotion states by reconfiguring to expose different functionality of the robot. Reconfiguration enables Scorpio to easily expose the robot’s required functionality, which is otherwise difficult or not possible. Some examples of a commercial reconfigurable robots are hTetro [11], hTrihex [12], and hTetrakis [13]. Like other autonomous indoor vacuum cleaners, hTetro can autonomously navigate to clean the indoor environment. However, because of its self-reconfigurable ability, it is able to change its shape to reach tight places where a normal fixed-shaped autonomous vacuum cleaner is unable to. This allows hTetro to achieve greater performance than its competitors.

Self-reconfigurable robots is a broad field and there are previous works to classify the types of self-reconfigurable robots to understand the exact type that self-reconfigurable robots fall under. In the works of [14,15], there are two types of self-reconfigurable robots. The first is a robot that comprises a group of intelligent modules, and the second is a robot that comprises of a group of modules where the intelligence comes from an external agent. In our previous work, we have classified self-reconfigurable robots into three categories which are nested reconfigurability [16,17,18], intra reconfigurability [19,20], and inter reconfigurability [21,22]. The hTetro described earlier has nested reconfigurability. In this paper, the robot that we will be focusing on is a pavement sweeping robot with an intra reconfigurability where intelligence is drawn from an outside agent.

The current state-of-the-art autonomous mobile robots have control systems and perception systems to enable the robot to understand the surroundings and perform controlled movements in response. There are many different types of control systems used for different applications of autonomous mobile robots. The proportional integral and differential (PID) controller is one of the most common and stable algorithms used [23], and it is used in many applications, including autonomous cars. Other types of controllers include Model Predictive Controls [24], which optimizes the parameters to control the robot and requires higher computing power. There are many other controllers, such as the path tracking [25], which can be used with PID parameters to control speed, fuzzy logic with PID control [26] that can adaptively change its PID constant values, and adaptive PID control, which continuously updates its PID constants. The pavement sweeping robot Panthera, which consists of eight wheel motors, two scrubbing wheel motors, and one lead screw motor for locomotion and reconfiguration, is controlled using the PID controller computing the error between the input speed and actual speed of each of the motors. In order to achieve an autonomous reconfigurable robot, the robot has to understand the environment based on the perception sensors and then perform feedback control to the robot’s locomotion and reconfiguration units, and human localization with sensor feedback [27], so that the robot can operate in the environment smoothly and safely.

A pavement sweeping reconfigurable robot is designed to adaptively reshape to maneuver past such narrow pavements, wven when pedestrians use the pavement. To do this, the reconfigurable robot must understand its surroundings and other pavement users, such as pedestrians [28].

In addition, it requires the robot to understand how fast it must reconfigure and which mode of reconfiguration is required. Two aspects are required for a pavement sweeping robot to operate with a pedestrian: to have a robust control system for the robot kinematics and to have a perception feedback algorithm to the robot kinematics and control. However, there have been little works on pavement sweeping reconfigurable robots and vision perception algorithms for reconfigurable robots to perform locomotion and reconfiguration based on their surroundings. We had previously performed a study [29] for developing synergy with dynamic changing pavement widths and they are currently working to develop more robust vision perception algorithms to include more scenarios to enable Panthera to be aware of the pedestrian. Although our previous work enables the pavement sweeping reconfigurable robot to adapt to changing pavement widths, it is in a highly constrained situation where there are no pavement users during reconfiguration and that the change in pavement width is symmetrical. It is unable to accommodate pavement users and non-symmetrical pavement shapes in its feedback control from the vision sensor.

Multiple approaches have been proposed to understand the surroundings contexts which are presented surrounding mobile autonomous robots. Most of these approaches use image sensors such as Red Green Blue (RGB) camera sensors [30], depth sensors, and Light and Detection Ranging (LiDar) sensors. With advances in computer vision, perception algorithms, and robust control of the robot, an autonomous reconfigurable robot will be able to operate with good synergy with the surroundings. One common approach uses a combination of sensors to understand the environment in the form of sensor fusion. Many features of the surroundings can be derived using an RGB camera and a depth camera. In the past, computer vision engineers have used feature detection to manually look for features in the image to identify the objects in the surroundings. One example is the canny edge detection [31], where the algorithm looks for lines in an image to identify possible features, such as road lanes. Although they work well in a friendly road setting, they do not perform very well when lanes are obstructed by obstacles, such as another car. Another possible issue is that different lightings will affect the accuracy of the Canny edge detection. However, there are algorithms to reduce image dependency on lightings, such as converting RGB images to HLS or HSV, new techniques of camera fusion, and deep learning [32] achieve better results. Over the past decade, many kinds of research have gone into developing learning algorithms that enable the computer to learn a model and predict accurately due to the surge of autonomous cars and robots, one field that is growing rapidly in the field of deep learning convolutional neural networks (DLCNN). DLCNN [33] are models that enable the algorithm to automatically learn the features of an image. With deep learning approaches, it is able to compute even unique features that are not able to be perceived by humans. Hence, deep learning performs much better than traditional computer vision techniques, such as scale-invariant feature transform and feature-based Histogram of Orientation. Although deep learning such as Reinforcement Learning [34] is able to perform much better than traditional computer vision techniques and algorithms, it comes at a much higher computational cost and requires much higher processing power. Advances in technology also led to better computers that are able to compute high processing power. New parallel computing platforms, such as Compute Unified Device Architecture (CUDA) [35] created by Nvidia, enable high-level processing to be done in a shorter period. With the development of NVIDIA CUDA Deep Neural Network [36], highly tuned implementations for forward and backward propagations are required in deep learning network models, allowing computation can be done on GPU at a much faster rate. This allows robots to perform high-level processing that was not possible in the past. Furthermore, with the rise of connectivity and the Internet of Things [37], where hardware is integrated into the cloud computing [38], robots can outsource high computational power requirements to commercial cloud platforms. Some examples are Alibaba Cloud, Kamatera, and DigitalOcean. Deep learning involving high-level computational power is now feasible and it can even be performed in real-time.

A comprehensive description of a reconfigurable pavement sweeping robot, Panthera, will be specified in this paper. Based on Panthera’s design, it can change the size of its width while moving; it also has the ability to perform zero radius turn, moving sideways, and forward and reverse motion. With robust control and a perception feedback control, Panthera will perform cleaning with lesser disruption to the pedestrian. Using encoder sensors that are attached to the motors, feedback control of the motor speeds can be developed. Together with Panthera’s kinematics model, reconfiguration during locomotion can be performed smoothly in a controlled manner. Using RGB and Depth cameras, sensor fusion, and new methods of deep learning, semantic segmentation, Panthera will aware of pedestrians in its surroundings and perform locomotion and reconfiguration changes to reduce disruption to pedestrians. Taking into account the purpose of developing a higher level of autonomy for Panthera, the paper presents the following objectives: (1) to develop a robust kinematics model for reconfiguration during locomotion, (2) to develop vision-based perception algorithm to understand the pedestrian speed and position, and (3) to develop a vision based kinematics control that is based on pedestrian speed and position.

The paper will be structured into the following sections. Section 2 provides a comprehensive understanding of the design of the sweeping pavement robot Panthera. Section 3 describes the use of pavement width and pedestrian density reconfiguration. Section 4 provides Panthera’s kinematics model. Section 5 provides the experimental results of the kinematic control performance. Section 6 provides a summary and discusses future works.

## 2. Robot Architecture

This section will discuss the design principles and design layout of Panthera [39]. The definitions of the terminologies used can be found in Appendix A.

### 2.1. Design Principles

Ideally, spaces are designed for the robot, as discussed in [40]. However, many areas are not yet designed for robots. Therefore, a reconfigurable pavement sweeping robot has to be designed to overcome the pain points poses by the existing infrastructure, which are not well designed for robots. Existing cleaning vehicles and robots experience the pain points that are caused by different pavement types. Typical pavement types that pavement sweeping robot encounters can be seen in Figure 1. The pavement types are, as follows. Wide pavements, narrow pavements, converging and diverging of pavements, pavements with sharp and narrow turns, pavements with obstacles, and dynamically changing width pavements. Besides, there are pavement users, such as pedestrians and animals using the pavement. Existing cleaning vehicles can operate in wide pavements, but they are not able to operate in areas not designed for robots, such as tight spaces. Thus, a reconfigurable pavement sweeping robot is introduced to overcome difficulties that a traditional pavement sweeping robot cannot, and it will have the following abilities:

- Able to move laterally with four independent differential drive steering units.
- Able to perform very tight turns and static rotation.
- Able to perceive surrounding pavement widths and pedestrians with vision techniques
- Able to change the size of its width with a range from 70 cm to 200 cm.
- Able to carry up to 200 kg.

**Figure 2:**
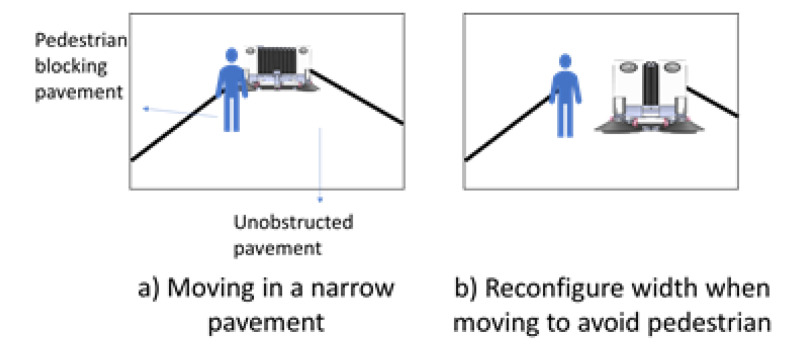
Panthera reconfiguration to avoid pedestrian while cleaning pavement.

Figure 2 demonstrates the notion of a pavement sweeping self-reconfigurable robot that is moving along a small width pavement while avoiding a pedestrian. Next, the design layout of the robot Panthera will be discussed.

### 2.2. Design Layout

**Figure 3:**
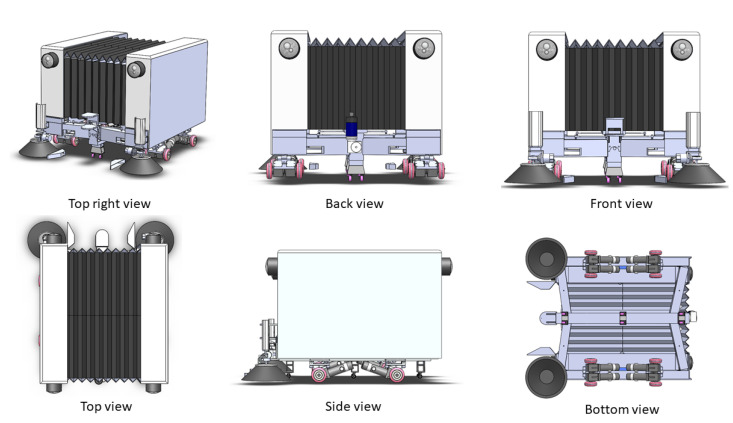
Panthera Body Structure.

The design of Panthera [41], as seen in Figure 3 will be categorized into Mechanical Design, Steering Unit Design, Electrical Design, Reconfiguration Design, and System Design.

#### 2.2.1. Mechanical Design

Panthera’s body structure is made of an aluminum frame, which is supported by four steering units. Each steering unit consists of a differential drive. The body’s aluminum frame consists of a central beam and two side beams together, which are joint with steel hinges and linkages. This forms a scissors mechanism that enables the robot body to reconfigure the width of the robot. A double-threaded lead screw has the dimensions of 32 mm in diameter and 10 mm in pitch and is connected to a Direct Current (DC) motor and flange bearing. The linear actuation that is caused by the lead screw rotation will translate to the side beams’ linear motion. This enables the lead screw DC motor to push and pull the side beams for Panthera’s body reconfiguration, which enables it to expand and contract. The aluminum frame is the structure that supports the batteries, electrical components, and two sweeping brushes, which are powered by two motors, respectively. The aluminum frame is hollow and it has dimensions of 8 mm in thickness. The Panthera aluminum frame is covered with a customized artificial leather bellow design and it has a dimension of 1.5 mm in thickness, enabling the cover to compress and expand together with the aluminum side beams. The cover protects the interior of Panthera from the environment. This keeps the electronics and mechanism free and protected from foreign objects, such as leaves, twigs, branches, and even splashes of water.

#### 2.2.2. Steering Unit Design

**Figure 4:**
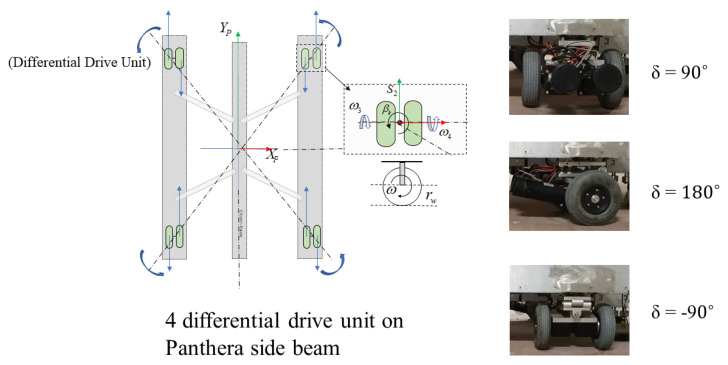
Differential Drive Steering Units.

Each steering unit consists of a two-wheel differential drive that is powered by two DG-158A 24 V motors. Each wheel is 108 mm in diameter. Panthera has four independent steering units $SU_{1}$, $SU_{2}$, $SU_{3}$, and $SU_{4}$. The steering units consist of a two-wheel differential drive, which helps to distribute the Panthera aluminum frame’s weight evenly. The steering units can perform a 360-degree rotation. Steering unit velocity and steering angle can be controlled independently, as seen in Figure 4. Because Panthera consists of four independent steering units, Panthera is able to perform movements, such as zero angle turning radius and moving sideways. Although the steering units are independent, they have to synchronize, depending on the locomotion and reconfiguration that are desired to avoid slipping or sliding the wheels.

#### 2.2.3. Electrical Design

**Figure 5:**
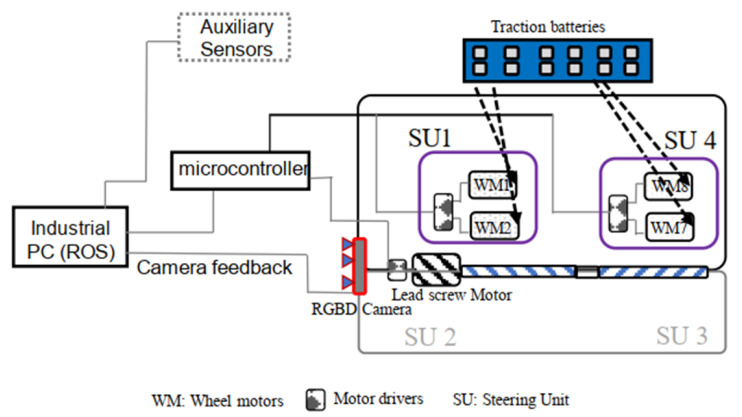
Electrical Layout.

Panthera is powered by a series of 12 2 V traction batteries. The resultant 24 V combined voltage power supply powers two 12 volt scrubbing wheel motors, eight DG-158A 24-volt wheel motors, and the 24 volt lead screw reconfiguration motor. The processing required for locomotion and vision feedback is computed on the mounted high specification computer system. A2 optical encoders are attached to the steering units to feedback the steering angle of the steering units, while incremental encoders are fixed at all motors for speed feedback on the actual speed of the motors. The incremental encoders and the corresponding motors that are connected directly to RoboClaws connected to an Arduino Mega 2560 for feedback control. A RealSense D435 camera, which is a Red Green Blue (RGB) and Depth camera, and the A2 Optical encoder is connected to a high specifications computer system that is capable of high-level processing. The onboard industrial computer system is run on Robot Operating System is directly connected to the Arduino microcontroller to communicate the vision perception output and the A2 Optical encoder data values for feedback control. The high-level electrical layout can be seen in Figure 5, where each steering unit consists of two DG-158A 24-volt wheel motors.

#### 2.2.4. Reconfiguration Design

**Figure 6:**
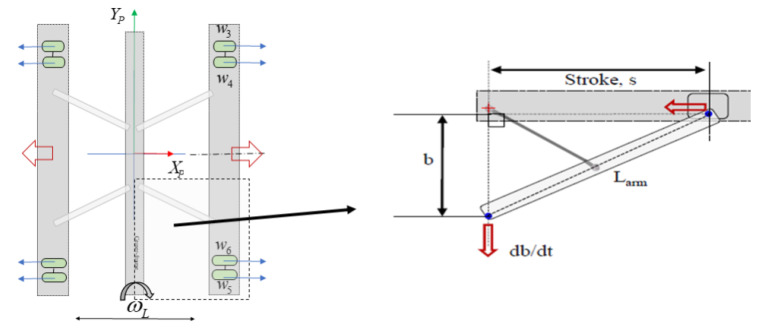
Reconfiguration Constrain.

The reconfiguration design of Panthera consists of its steering units, lead screw motor, and scissors mechanism. Because the lead screw pitch is 10 mm, one revolution of the lead screw translates to 10 mm linear actuation by the carriage. The linear actuation that is caused by the rotation of the lead screw will translate to the linear motion of the side beams, which are connected to the steering units. For a smooth reconfiguration, the scissor mechanism constraints shown in Figure 6 must be obeyed. The perception unit will send inputs to the steering units and lead screw motor, which obeys the scissor mechanism constrain, while the controller will ensure that the actual speed of the individual motors is maintained close to the input speed.

For a smooth reconfiguration, the speed of the steering units, the heading angle of the steering units, and the lead screw motor have to be synchronized and obey the scissor mechanism constraint in Figure 6. The perception unit will send inputs to the steering units and lead screw motor, which obeys the scissor mechanism constraint, while the controller will ensure that the actual speed of the individual motors is maintained close to the input speed.

#### 2.2.5. System Design

**Figure 7:**
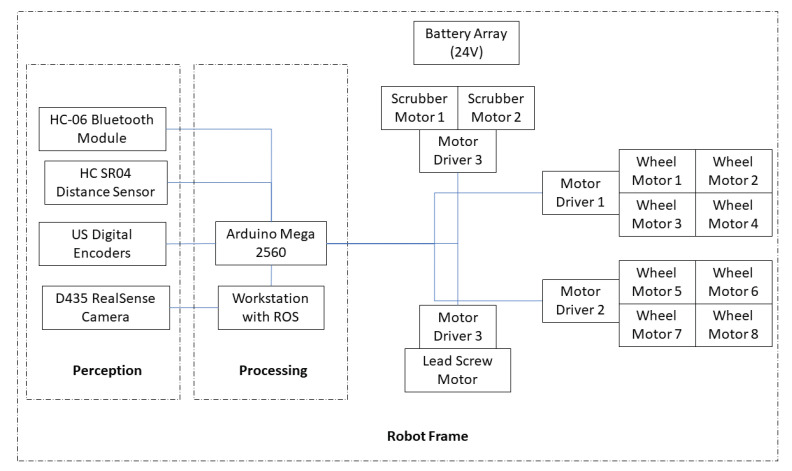
Panthera system architecture.

The Robot Operating System (ROS) is used as a middleware to enable data transmission and processing sensors for Panthera to perform locomotion and reconfiguration. The onboard high specification computer has eight CPU cores, 16 GB RAM, and a GPU card that enables high-level processing. The onboard computer serves as the ROS Master core, where nodes and topics are used for computation and communication. Figure 7 illustrates the system design. All of the motors, eight-wheel motors, two scrubbing wheel motors, and the lead screw motors are powered by a 24 V battery controlled by the Arduino Mega 2560 micro-controller. An RGB-D RealSense D435 camera is used as a perception unit to understand the environment and process it with the high specification onboard computer to extract essential information about the surroundings for feedback control. All of the data from the sensors and the processed perception unit output are transmitted through the ROS server to the micro-controller for locomotion and reconfiguration. This enables the robot to gather data of the surroundings through the sensors and process the data to understand the locomotion and reconfiguration desired in order to develop good synergy with its dynamically changing surroundings.

## 3. Pavement Aware Robot

This section will discuss the framework and methodology of a pavement aware robot.

### 3.1. The Framework of Pavement Aware Robot

Panthera has the ability to reconfigure in width based on the requirements demanded by the surroundings and users of the surroundings because Panthera has the mechanisms designed to reconfigure in width to tackle pain points that fixed-shaped pavement cleaning vehicles are unable to perform. Therefore, it has to collect data from the surroundings and make sense of the data in terms of locomotion and reconfiguration during its cleaning operations. The Panthera perception unit will employ a RealSense D435 RGB-D camera and process the stream of images while using the deep learning convolutional neural network to derive the locomotion and reconfiguration required. Because Panthera is a pavement sweeping robot, Panthera has to understand the pavement that it is in, and will do so by understanding the pavement widths. It also has to understand the pedestrian density on the pavement that Panthera is in. From the RGB-D image, Panthera will estimate the pavement widths. Furthermore, Panthera is able to identify pedestrians on the pavement and estimate the distance the pedestrian is away from Panthera, the pedestrian relative velocity, and space that the pedestrians occupy. From the estimated pavement width, estimated space the pedestrians occupy, pedestrian relative velocity, and the depth values, Panthera is able to understand the surroundings that it is operating in and derive two steering angles for the steering units, beta left and beta right, and perform locomotion and reconfiguration that is required from the surroundings. Beta left is the steering angle for steering unit 1 and 4, while beta left is the steering angle for steering unit 2 and 3 required for Panthera locomotion and kinematics. Beta left and beta right act as key parameters that enable Panthera to reconfigure and accommodate pavement users and non-symmetrical pavement shapes during its cleaning operation. Based on the derived steering angles from the perception unit, Panthera will be able to calculate the steering unit and lead screw required speed for locomotion and reconfiguration. The proposed algorithm assumes that Panthera is parallel to the pavement.

### 3.2. Methodology of Pavement Aware Robot

Many types of research have been invested in the development of perception algorithms in the past two decades. Especially in the field of autonomous cars, pavement width estimation has been extensively studied. However, there are little works on using pavement widths estimation to understand the changes in pavement widths for the reconfiguration of a pavement sweeping robot. This involves using perception sensors to understand the pavement and giving feedback to a reconfigurable robot’s kinematics model to perform reconfiguration. Traditional computer vision techniques were widely popular and used to detect the edges of lanes in autonomous cars. However, in non-ideal situations, such as when there are no proper road signs or road dividers, it does not perform well. Improvements in technology have developed newer methods to detect roads and pavements for width estimations. One example is semantic segmentation. Semantic segmentation uses the deep learning convolutional neural network (DLCNN) to classify pixels and localize pixels in an image. Even the current state-of-the-art autonomous mobile robots uses semantic segmentation for their autonomy. Using newer DLCNN approaches, we can classify pavements and localize the pixels, even when pavements do not have proper and clearly defined markings and outperform traditional feature detection methods. However, the challenge with using DLCNN is the effort that is required in collecting quality data to train the model and the high computing power that is required to run the model in real-time.

**Figure 8:**
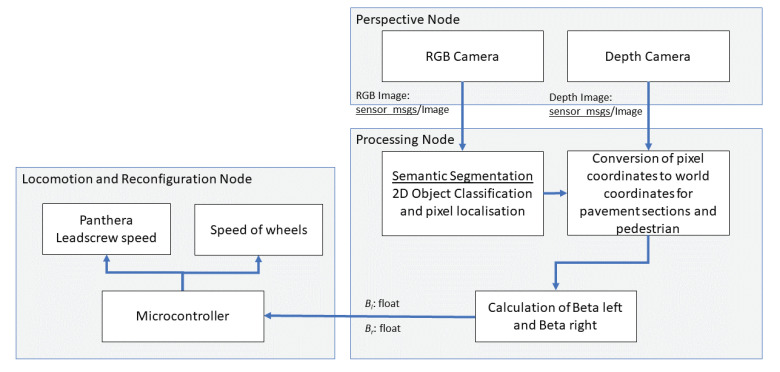
The block diagram of vision-based reconfigurable mechanism.

Advances in research have led to the development of readily available and pre-trained deep learning models, such as Deeplab [42] and Segnet [43], which are capable of semantic segmentation at a lower computational cost and can be implemented in real-time. Many other semantic segmentation performs the classification of objects and localization of the respective pixels with high accuracy and in real-time, such as Fast-SCNN [44], and FasterSeg [45]. Using Panthera’s high specification onboard computer, Panthera will process the stream of images from the RealSense D435 camera in real-time. The onboard computer runs on the Robot Operating System (ROS) and it has the framework to allow real-time communication between sensors, the onboard computer, and the Arduino. Using the ROS framework, the onboard computer is able to publish and subscribe topics to ROS nodes in parallel. ROS nodes are a computational process that that subscribes or publish information through the ROS topic. This allows for Panthera to perform its data transfer, feedback control, and perception unit in parallel. Figure 8 shws the architecture of the ROS node diagram. In the perspective node, the RealSense D435 camera will publish the live stream of RBG-D sensor image messages via a topic. The processing node will subscribe to the topic, which is published by the perspective node. In the processing node, semantic segmentation will be performed to classify the objects in the image and localize the classified pixels. From the localized and classified pixels, we will be able to better estimate the pedestrian’s distance [41] and relative velocity. The pedestrians’ distance is determined by the averaged depth values of the pixels that are labeled as pedestrians, and the instance relative velocity vector of the tracked pedestrians is determined from the differential of the distance of pedestrians based on a 0.2 s time step. Besides, with the localized and classified pixels, we can determine how the pavement’s width in the two sections of the RGB stream images enables the vision unit to derive two parameters $\beta_{l}$ and $\beta_{r}$, which feedbacks to the Panthera control system. The vision unit feedback control allows for Panthera to perform locomotion and reconfiguration based on the dynamically changing pavement width. We did propose an improved vision algorithm of [29], which enables Panthera to avoid moving pedestrians and, at the same time, reconfigure its width concerning the dynamically changing pavement widths.

For Panthera to operate in a dynamically changing pavement width environment with pavement users, Panthera has to reconfigure in size before a pedestrian, who is walking towards Panthera, reaches Panthera and, at the same time, continuously adapting to varying pavement widths. To achieve this, Panthera needs to start reconfiguration, so that, when the pedestrian reaches Panthera, Panthera will be in the reconfigured state, and the pedestrian will not need to disrupt their walking. Panthera will constantly track the distance the pedestrian is away from Panthera and the pedestrian’s relative velocity. It is assumed that the pedestrian is walking in a straight path towards Panthera. Based on Panthera current speed, *v*, and the relative pedestrian speed, $v_{p}$, and the distance between Panthera and the pedestrian, $d_{p}$, the position where the pedestrian and Panthera meet, $d_{position}$, can be calculated, as follows:

(1) $$d_{position}=d_{p}\frac{v}{v_{p}}$$

After the semantic segmentation of the RGB images, Panthera is able to determine the pixel coordinates of the pedestrians and the corresponding pavement edges. The center x pixel coordinate, $x_{cp}$, of the pedestrian, is used to derive an estimation of the pedestrian’s location. As the pedestrian is walking straight into Panthera without changing their direction, the pedestrian center x pixel, $x_{cp}$, coordinate of the pedestrian should not change much at position $d_{position}$, and the minimum, $Min(w_{p})$, and maximum, $Max(w_{p})$, x world coordinates of the pedestrian can be derived by adding or subtracting a 0.5 m offset of the width of a pedestrian. The average minimum, $x_{il}$, and maximum pixel, $x_{ir}$, x coordinate values for pavement edges are used for estimating the position of the pavement edges in two sections of the image [29]. Upon obtaining the pixel coordinates, $w_{2l}$, $w_{2r}$, $w_{1l}$, and $w_{1r}$ will be derived using Equation (4). Upon deriving pedestrian and pavement edges localized pixel coordinates, the processing node will subscribe to the depth images from the topic published by the perspective node. Because RealSense D435 is a stereo camera with both an RGB image sensor and a Depth image sensor, they are of the same resolution, and each pixel in the RGB image corresponds to the pixel in the Depth image. This enables us to map the pedestrian and pavement edges localized pixel coordinates to their depth value. When the depth images are subscribed, the image will be filtered via an adaptive directional filter [46] to remove noise. After filtering the depth image, the filtered depth image will be used to derive the depth values that are based on the pedestrian and pavement edges of localized pixels. The depth value of each pavement section, $d_{i}$, is then computed, as follows:

(2) $$d_{i}=\frac{\sum_{\Omega_{i}}d_{k}}{\Omega_{i}}$$

where $\Omega_{i}$ represents the sum of pixels categorized as pavement in section *i*, and $d_{k}$ represents the value of the depth in the pixel. The depth value of the pavement section is the average of the depth pixel value that is categorized as pavement.

**Figure 9:**
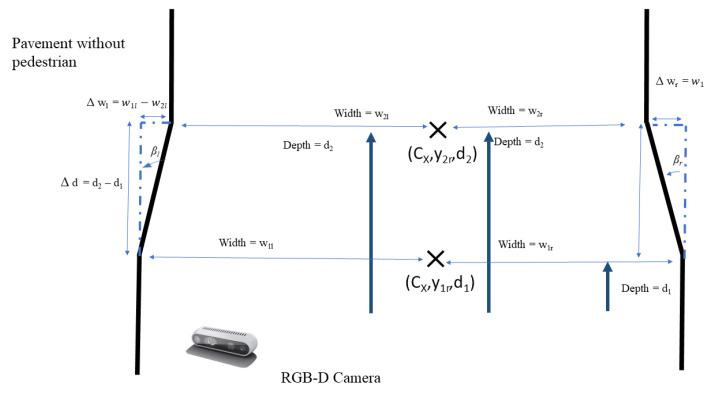
Panthera vision feedback adapting to pavement change concept.

**Figure 10:**
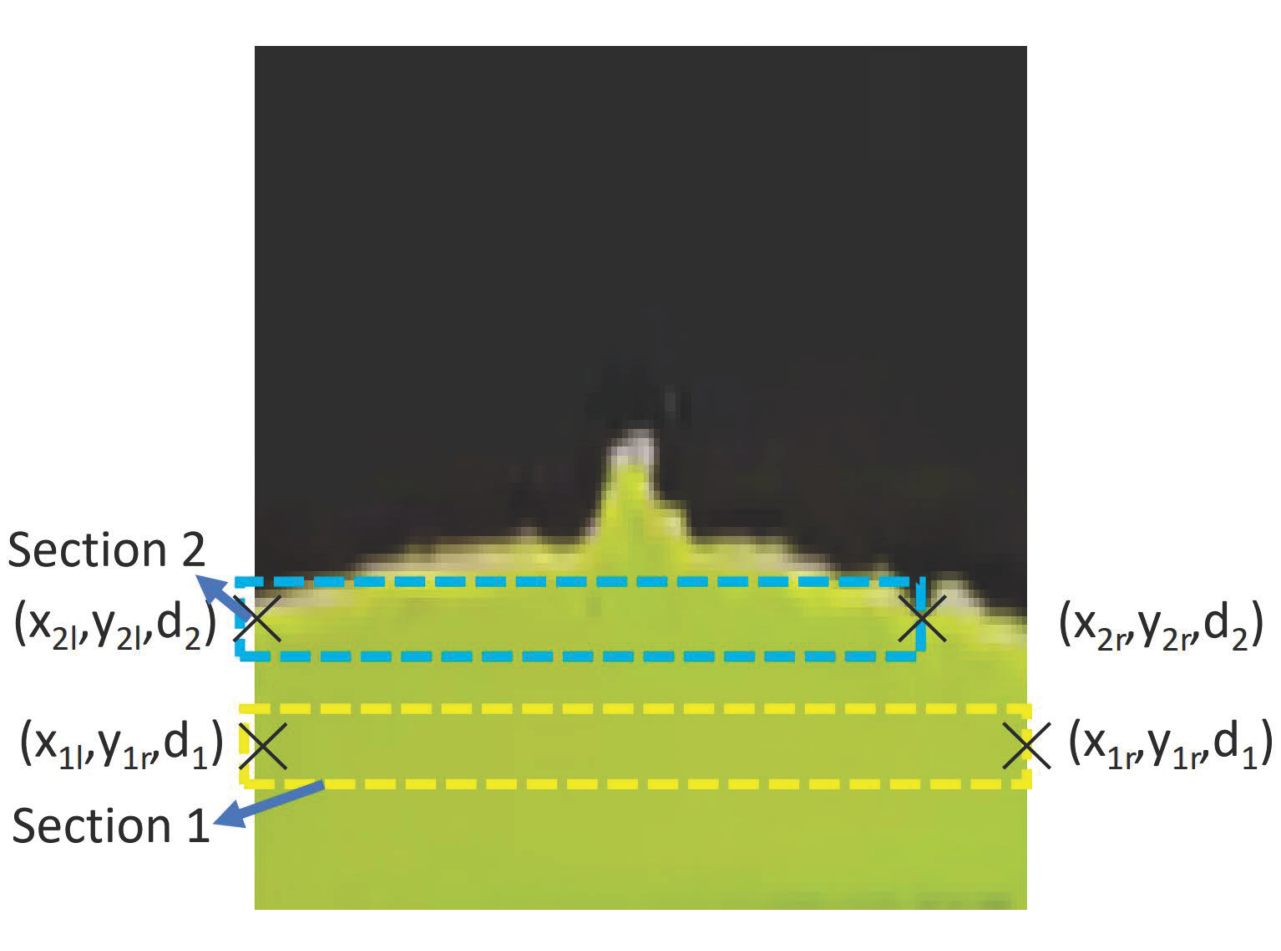
Pavement edge detection using semantic segmentation for two sections.

The depth value of the pedestrian, $d_{p}$, can be derived by the equation, as follows:

(3) $$d_{p}=\frac{\sum_{\zeta }d_{i}}{\zeta }$$

where ζ represents the sum of pixels that are categorized as pedestrian and $d_{i}$ represents the value of the depth in the pixel. The real world width values of $w_{2l}$, $w_{2r}$, $w_{1l}$, and $w_{1r}$, and pedestrian real-world coordinates, $w_{p}$, will be estimated by conversion of x and y pixel coordinate system to x and y world coordinate system. The RealSense RGB-D sensor has in-built functions to convert the x and y pixel coordinate system to the x and y world coordinate system. This is done through camera calibration techniques. Camera intrinsic and extrinsic matrix can be obtained using camera calibration methods. Camera calibration methods give us the focal length, $f_{x}$ and $f_{y}$, of the RealSense RGB-D sensor and the optical center, $c_{x}$ and $c_{y}$, of the RealSense RGB-D sensor in its respective x and y coordinate to obtain the intrinsic camera matrix, *I*. The RealSense RGB-D sensor in-built functions also provide us with the translation vector, *K*, and rotation vector, *Q*. The extrinsic matrix, *S*, can be derived where S=[QK]. We can convert from the pixel coordinate system to the world coordinate system, as follows:

(4) c=I∗[QK]∗F=I∗S∗F.

where *F* is the world coordinates and *c* is the pixel coordinates. When there are no pedestrians, the processing node uses geometry, as seen in the Figure 9, to calculate and publish $\beta_{l}$ and $\beta_{r}$. The locomotion and reconfiguration node will then subscribed the $\beta_{l}$ and $\beta_{r}$. When there are no pedestrian, only the difference in pavement widths, $\Delta w_{l}$ and $\Delta w_{r}$ is calculated from the four pavement width estimated, $w_{2}l$, $w_{2}r$, $w_{1}l$ and $w_{1}r$, where $\Delta w_{l}=w_{1l}-w_{2l}$ and $\Delta w_{r}=w_{1r}-w_{2r}$ as seen in Figure 10. Similarly, Δd is calculated from the two pavement depth values, $d_{1}$ and $d_{position}$, where $\Delta d=d_{2}-d_{1}$. From Δd, $\Delta w_{l}$, and $\Delta w_{r}$, the values $\beta_{l}$ and $\beta_{r}$ can be derived, as follows:

(5) $$\beta_{l}=\arctan\frac{\Delta w_{l}}{\Delta d}$$

(6) $$\beta_{r}=\arctan\frac{\Delta w_{r}}{\Delta d}$$

**Figure 11:**
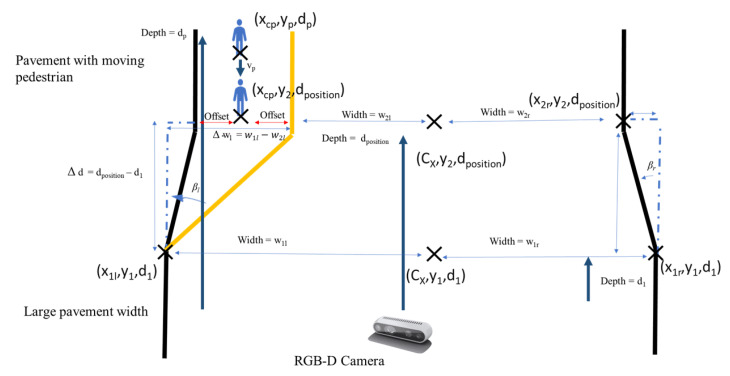
Panthera feeback to avoid pedestrians while adapting to pavement change concept.

**Figure 12:**
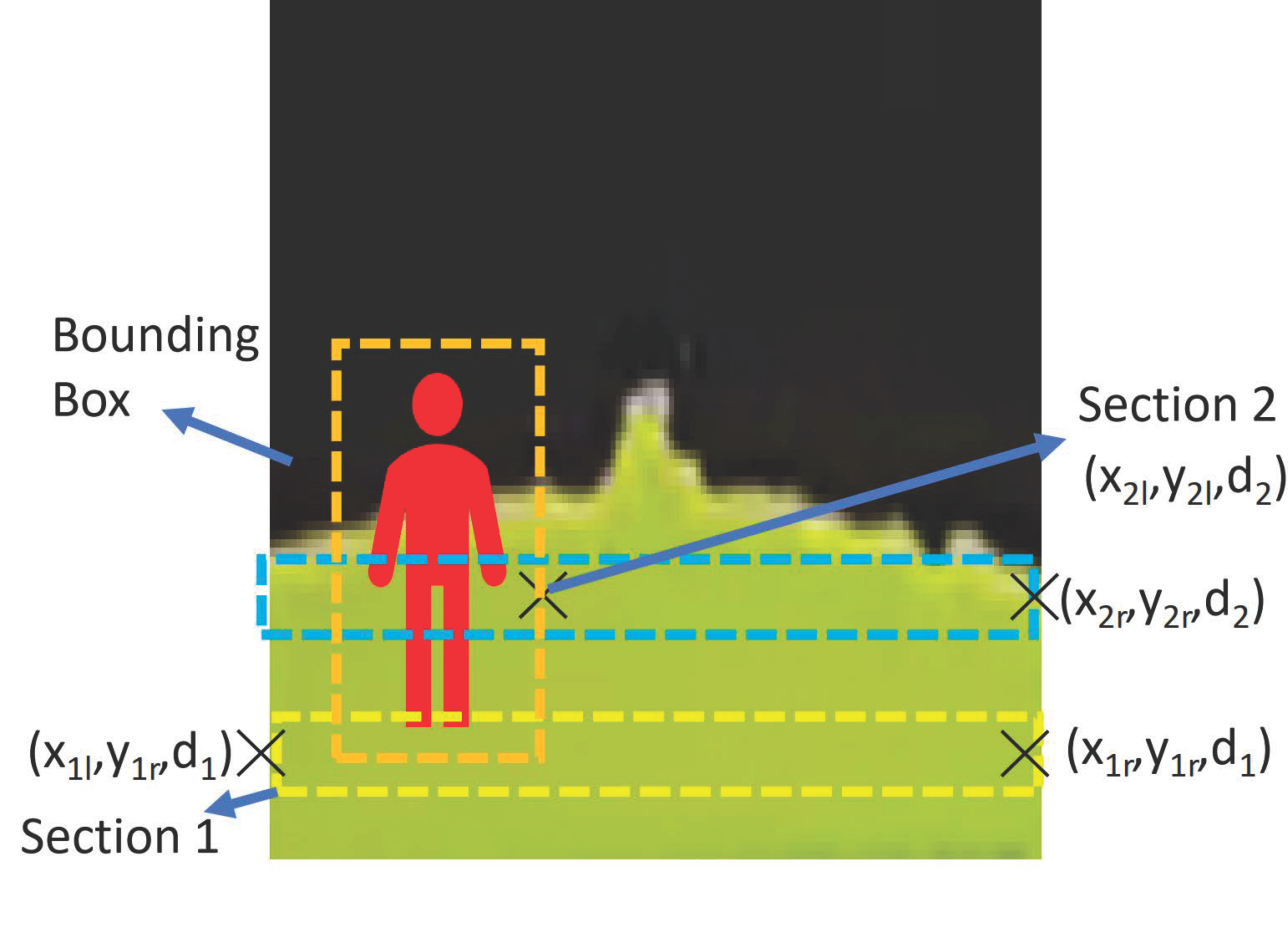
Panthera feedback to avoid pedestrians while adapting to pavement change.

However, when a pedestrian is detected, the processing node will perform the algorithm, as seen in the Figure 11, to calculate and publish $\beta_{l}$ and $\beta_{r}$. The locomotion and reconfiguration node will subscribe the $\beta_{l}$ and $\beta_{r}$. When there is a pedestrian, the pedestrian center x pixel, $x_{cp}$, will be converted to the real world pedestrian coordinate, $w_{p}$, first. The minimum and maximum x world coordinate of the pedestrian will be calculated while using the equation $Min\left(w_{p}\right)=w_{p}-$ offset and $Max\left(w_{p}\right)=w_{p}+$ offset where the offset is set to 0.5 m. $w_{2l}=\left|Max\left(w_{p}\right)\right|$ and $w_{2r}=\left|Min\left(w_{p}\right)\right|$ when the pedestrian is on the left or right side of the pavement respectively. The $w_{2l}$ and $w_{2r}$ on the side without the pedestrian are the world coordinates of the pavement edges at the depth value $d_{position}$. When there is a pedestrian detected, $\Delta d=d_{position}-d_{1}$, $\Delta w_{l}=w_{1l}-w_{2l}$ and $\Delta w_{r}=w_{1r}-w_{2r}$ as seen in Figure 12. From Δd, $\Delta w_{l}$ and $\Delta w_{r}$, the values $\beta_{l}$ and $\beta_{r}$ can be derived using (5) and (6).

$\beta_{l}$ and $\beta_{r}$ derived from the processing node will be continuously published as a topic to the locomotion and reconfiguration node for the visual feedback of the Panthera surroundings, so that Panthera will constantly know how the pavement width is changing and it will be able to change its shape, as required by the surroundings. The next section will discuss the kinematics and control of Panthera.

## 4. Kinematics and Control

This section will discuss the kinematics for reconfiguration and the control model used to enable the reconfiguration to be performed with stability.

### Control System

**Figure 13:**
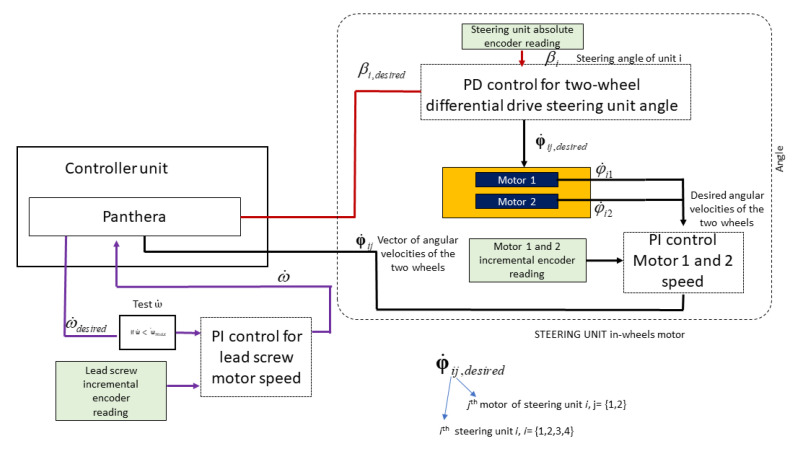
Control Model Layout.

A robust control model is vital for Panthera locomotion and reconfiguration to perform safely during operation and ensure that the mechanical constraint is always adhered to. Similar to many commercial robots, the Proportional Integral and Differential (PID) controller is employed for ensuring that the speed of all Panthera’s motors are working as desired. PID controller is a control loop that takes the proportional, integral, and differential of the error between the actual value and desired value for system control. The PI controller is applied for all motors to ensure that the motors’ speed is similar to the speed of the input desired by the kinematics. Another PD controller is applied for steering control to ensure that the steering angles are similar to the steering angle input desired from the kinematics model. Figure 13 shows the control system of Panthera.

As the steering unit consists of a two-wheel differential drive, the steering unit in-wheels controller will take in the desired steering angle. Based on the difference between the encoder readings and desired angle, it will perform the PD controller in order to adapt the left and right wheel angular velocity to perform differential steering. The angular velocity for motor 1 and motor 2 will be controlled based on PI, as seen in Figure 13.

The lead screw reconfiguration motor has a maximum speed, $\omega_{max}$ of about 53,000 quad pulses per second (QPPS). The lead screw carriage has a movable range, *d*, of 510 mm, and the full encoder count, Enc, for the movable range is 1,625,109 quad pulses. The derived reconfiguration motor speed ω, from the reconfiguration kinematics is Equation $\omega_{ls}=\frac{\dot{s}d}{Enc}$, where $\dot{s}$ is the velocity of the carriage that can be derived from Equation (21).

Depending on the input differential steering unit speed $v_{i}$, $\omega_{ls}$ may exceed $\omega_{max}$. In order to overcome the constrain, when $|\omega_{ls}|$>$|\omega_{max}|$, $\omega_{desired}$ is set to $\omega_{max}$ and, $v_{yi}$ and φ has to be derived from the inverse kinematics from the above equations. For static reconfiguration, a drop in $v_{yi}$ results in a drop in $v_{i}$, while, for the reconfiguration while moving, a drop in $v_{yi}$ results in the change of the steering angle, $\beta_{i}$.

Proportional Integral (PI) feedback control controls the wheel motor speed and the reconfigurable motor speed. Proportional Differential (PD) feedback control is used for steering control. The input speed will be given to the reconfiguration motors and wheel motors, and an encoder will read the actual speed values of the respective motors and send them as feedback to the controller unit. The inbuilt PI controller unit in RoboClaw 2X15A is used to perform the feedback. Figure 13 shows the control model.

## 5. Kinematics Overview

Because Panthera is an eight-wheel drive with four steering units and it has a reconfiguration mechanism, limited works have been done on this type of robot’s kinematics. In this chapter, we will study, in detail, the locomotion kinematics and reconfiguration kinematics of Panthera to enable it to perform the objective of developing a robot that can reconfigure in width while it is moving.

## 6. Locomotion Kinematic Model

**Figure 14:**
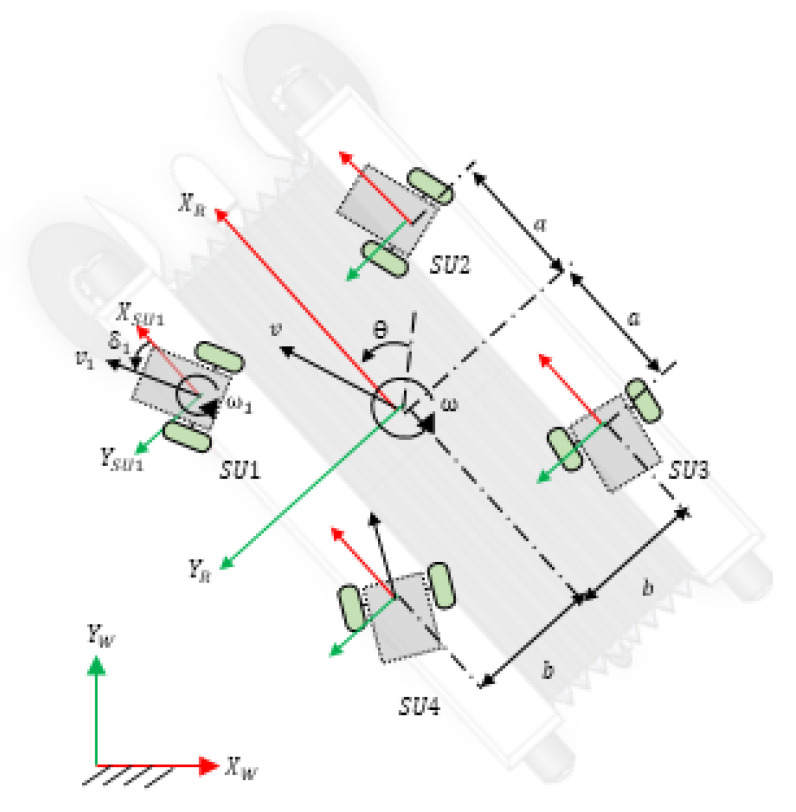
Robot reference frame.

There are four steering units ($SU_{1}$, $SU_{2}$, $SU_{3}$, and $SU_{4}$) in Panthera, as seen in Figure 14. The coordinates of the steering units are defined as $\left(x_{SU1}^{R},y_{SU1}^{R}\right)=\left(a,b\right)$; $\left(x_{SU2}^{R},y_{SU2}^{R}\right)=\left(a,-b\right)$; $\left(x_{SU3}^{R},y_{SU3}^{R}\right)=\left(-a,-b\right)$; $\left(x_{SU4}^{R},y_{SU4}^{R}\right)=\left(-a,b\right)$. where a=0.335 m and 0.19 m ≤ b ≤ 0.65 m. The robot is fully closed when b = 0.19 m and fully expanded when b = 0.65 m. *v* is linear velocity of the robot and $v_{i}$ is the velocity of the steering unit, $SU_{i}$. $v_{x}$ and $v_{y}$ are the robot velocity component along $X_{R}$ and $Y_{R}$ axis, respectively, and obeys the following equation $v=\sqrt{{v_{x}}^{2}+{v_{y}}^{2}}$, $v_{i}=\sqrt{{v_{xi}}^{2}+{v_{yi}}^{2}}$. The steering unit velocity component along the $X_{SUi}$ and $Y_{SUi}$ axis are $v_{xi}$ and $v_{yi}$ respectively. Because Panthera is required to move smoothly, it should not experience any wheel slipping or sliding. Based on the constraint equation of no slipping and no sliding, the velocity of the robot along the $X_{SUi}$ and $Y_{SUi}$ and its respective steering units are related, as follows:

(7) $$v_{xi}=v_{i}\cos\delta_{i}=v_{x}-y_{SUi}^{R}\omega$$

(8) $$v_{yi}=v_{i}\sin\delta_{i}=v_{y}-x_{SUi}^{R}\omega$$

where $\delta_{i}$ is defined as the anti-clockwise angle between the direction of $v_{i}$ and $X_{SUi}$, while $y_{SUi}^{R}$ and $x_{SUi}^{R}$ are the coordinates of *i*th steering units in the robot coordinate frame and ω is the angular velocity of the robot.

From Equations (7) and (8), the relationship between the velocities of the steering units, $v_{i}$, the velocity of the robot, $v_{x}$ and $v_{y}$, and the angular velocity of the robot ω can be derived, as follows:

(9) $$A\left[\begin{array}{c} v_{x}\\ v_{y}\\ \omega \end{array}\right]=B\left[\begin{array}{c} v_{1}\\ v_{2}\\ v_{3}\\ v_{4} \end{array}\right]$$

$$A=\left[\begin{array}{ccc} 1 & 0 & -b\\ 0 & 1 & a\\ 1 & 0 & b\\ 0 & 1 & a\\ 1 & 0 & b\\ 0 & 1 & -a\\ 1 & 0 & -b\\ 0 & 1 & -a \end{array}\right]$$

$$B=\left[\begin{array}{cccc} cos(\delta_{1}) & 0 & 0 & 0\\ sin(\delta_{1}) & 0 & 0 & 0\\ 0 & cos(\delta_{2}) & 0 & 0\\ 0 & sin(\delta_{2}) & 0 & 0\\ 0 & 0 & cos(\delta_{3}) & 0\\ 0 & 0 & sin(\delta_{3}) & 0\\ 0 & 0 & 0 & cos(\delta_{4})\\ 0 & 0 & 0 & sin(\delta_{4}) \end{array}\right]$$

Multiplying the pseudo-inverse matrix of A, $A^{+}$, to Equation (9) gives,

(10) $$\left[\begin{array}{c} v_{x}\\ v_{y}\\ \omega \end{array}\right]=A^{+}B\left[\begin{array}{c} v_{1}\\ v_{2}\\ v_{3}\\ v_{4} \end{array}\right]$$

where

(11) $$A^{+}=\left[\begin{array}{cccccccc} \frac{1}{4} & 0 & \frac{1}{4} & 0 & \frac{1}{4} & 0 & \frac{1}{4} & 0\\ 0 & \frac{1}{4} & 0 & \frac{1}{4} & 0 & \frac{1}{4} & 0 & \frac{1}{4}\\ \frac{-b}{z} & \frac{a}{z} & \frac{b}{z} & \frac{a}{z} & \frac{b}{z} & \frac{-a}{z} & \frac{-b}{z} & \frac{-a}{z} \end{array}\right]$$

and $z=4a^{2}+4b^{2}$.

Upon simplifying,

(12) $$\left[\begin{array}{c} v_{x}\\ v_{y}\\ \omega \end{array}\right]=\left[\begin{array}{cccc} \frac{cos(\delta_{1})}{4} & \frac{cos(\delta_{2})}{4} & \frac{cos(\delta_{3})}{4} & \frac{cos(\delta_{4})}{4}\\ \frac{sin(\delta_{1})}{4} & \frac{sin(\delta_{2})}{4} & \frac{sin(\delta_{3})}{4} & \frac{sin(\delta_{4})}{4}\\ K_{1} & K_{2} & K_{3} & K_{4} \end{array}\right]\left[\begin{array}{c} v_{1}\\ v_{2}\\ v_{3}\\ v_{4} \end{array}\right]$$

where $K_{i}=\left(-y_{SUi}^{R}cos\left(\delta_{i}\right)+x_{SUi}^{R}sin\left(\delta_{i}\right)\right)/z$.

Including the steering angle of the steering units, the state vector of the robot representing the robot position and orientation in the global coordinate frame is given to be **q** = ${\left[\begin{array}{ccccccc} x & y & \theta & \delta_{1} & \delta_{2} & \delta_{3} & \delta_{4} \end{array}\right]}^{T}$.

The kinematic model is then represented, as follows.

(13) $$\dot{q}=\left[\begin{array}{c} \dot{x}\\ \dot{y}\\ \dot{\theta }\\ \dot{\delta_{1}}\\ \dot{\delta_{2}}\\ \dot{\delta_{3}}\\ \dot{\delta_{4}} \end{array}\right]=\left[\begin{array}{cccccccc} \frac{cos(\delta_{1}+\theta )}{4} & \frac{cos(\delta_{2}+\theta )}{4} & \frac{cos(\delta_{3}+\theta )}{4} & \frac{cos(\delta_{4}+\theta )}{4} & 0 & 0 & 0 & 0\\ \frac{sin(\delta_{1}+\theta )}{4} & \frac{sin(\delta_{2}+\theta )}{4} & \frac{sin(\delta_{3}+\theta )}{4} & \frac{sin(\delta_{4}+\theta )}{4} & 0 & 0 & 0 & 0\\ K_{1} & K_{2} & K_{3} & K_{4} & 0 & 0 & 0 & 0\\ 0 & 0 & 0 & 0 & 1 & 0 & 0 & 0\\ 0 & 0 & 0 & 0 & 0 & 1 & 0 & 0\\ 0 & 0 & 0 & 0 & 0 & 0 & 1 & 0\\ 0 & 0 & 0 & 0 & 0 & 0 & 0 & 1 \end{array}\right]\left[\begin{array}{c} v_{1}\\ v_{2}\\ v_{3}\\ v_{4}\\ \omega_{1}\\ \omega_{2}\\ \omega_{3}\\ \omega_{4} \end{array}\right]$$

where $\dot{q}$ is defined as the velocity vector in the world coordinate.

Each steering unit is modeled as a differential drive robot, as illustrated in Figure 4. As the linear velocity of the right and left wheels vary, the steering unit will rotate about a point lying on the common left and right wheel axis, which is known as the instantaneous center of rotation (ICR). The ICR of each steering unit in their respective coordinate frame $SU_{i}$ is represented as $ICR_{i}=\left(-R\sin\delta_{i},R\cos\delta_{i}\right)$, where R is the radius of rotation curve from the ICR to the midpoint between the two wheels. ICR of each steering unit in the robot coordinate frame is then given by

(14) $$ICR_{i}^{R}=\left(x_{SUi}^{R}-R\sin\delta_{i},y_{SUi}^{R}+R\cos\delta_{i}\right)$$

Writing $ICR_{i}^{R}$ in the form of a vector ${\left[x_{ICRi}^{R}\;y_{ICRi}^{R}\right]}^{T}$ and applying a rotation matrix R(θ) to it, the ICR in the world coordinate frame is formed: $ICR_{i}^{W}={\left[x_{ICRi}^{W}\;y_{ICRi}^{W}\right]}^{T}=R^{-1}\left(\theta \right){\left[x_{ICRi}^{R}\;y_{ICRi}^{R}\right]}^{T}$[47], where $R^{-1}\left(\theta \right)$ is the rotation matrix given by

$$R^{-1}\left(\theta \right)=\left[\begin{array}{cc} cos(\theta ) & -sin(\theta )\\ sin(\theta ) & cos(\theta ) \end{array}\right]$$

The linear velocity of each steering unit in its respective coordinate frame is given by

(15) $$v_{i}=\left(v_{ri}+v_{li}\right)/2=\left(r{\dot{\varphi }}_{ri}+r{\dot{\varphi }}_{li}\right)/2$$

where $v_{ri},v_{li},{\dot{\varphi }}_{ri},{\dot{\varphi }}_{li}$ are the right wheel linear velocity, left wheel linear velocity, right wheel angular velocity, and left wheel angular velocity, respectively, and *r* is the radius of the wheel.

The angular velocity of each steering unit in its respective coordinate frame is given by

(16) $$\omega_{i}=\dot{\delta_{i}}=\omega_{ICRi}=\left(v_{ri}-v_{li}\right)/l=\left(r{\dot{\varphi }}_{ri}-r{\dot{\varphi }}_{li}\right)/l$$

where $\omega_{ICRi}$ is the angular velocity of the steering unit rotation about its ICR and *l* is the distance between the center of the right and left wheel.

The coordinates of the ICR of each steering unit in the robot coordinate frame can vary to produce a different mode of locomotion.

Case A: all $ICR_{i}^{R}$ = infinity or all $ICR_{i}^{R}$ = -infinity. The four steering units will be parallel to each other. This allows the robot to move in a straight line in any direction without changing its heading theta. The robot is said to be in Omni-directional mode.

**Figure 15:**
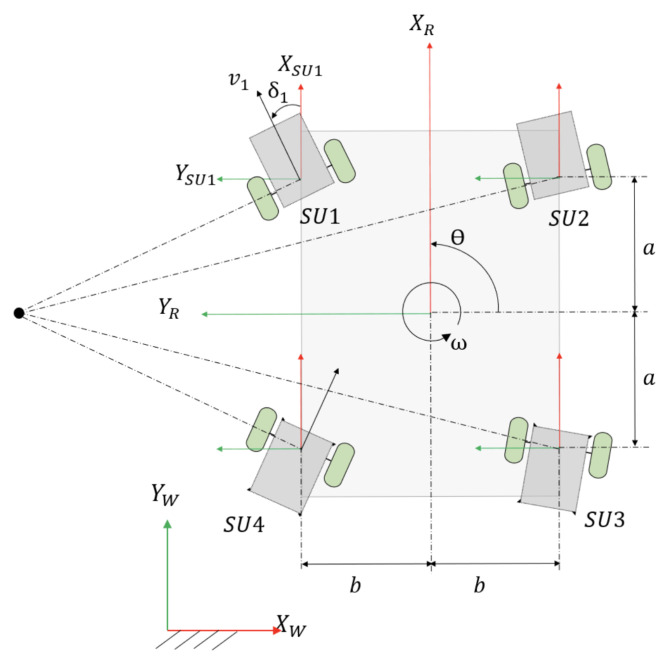
Common instantaneous center of rotation (ICR) of the four steering units.

Case B: $ICR_{1}^{R}=ICR_{2}^{R}=ICR_{3}^{R}=ICR_{4}^{R}=\left(k_{x},k_{y}\right)$, where $k_{x}$ and $k_{y}$ are positive or negative constants. The $ICR_{i}^{R}$ of the four steering units are the same, which allows the robot to rotate about this point, as shown in Figure 15. If $ICR_{i}^{R}$ has a positive y-coordinate, the robot turns left. If $ICR_{i}^{R}$ has a negative y-coordinate, then the robot turns right.

Case C: $ICR_{1}^{R}=ICR_{2}^{R}=ICR_{3}^{R}=ICR_{4}^{R}=\left(0,0\right)$ The ICR of all four steering units is at the centroid of the robot. The robot will perform a zero-radius turn without any translation motion.

Together with the lead screw motor, Panthera is capable of performing reconfiguration in width during locomotion. Reconfiguration requires the lead screw motor speed differential driving unit speed and steering angle to be synchronized. The locomotion and reconfiguration node will receive the pavement width estimates for the different sections from the processing node and then calculate the required lead screw motor speed, differential drive unit steering angle based on the Panthera locomotion. This allows for Panthera to reconfigure during locomotion at the rate that is required by the dynamic pavements and Panthera’s current speed.

### 6.1. Reconfiguration Kinematics

In order to perform reconfiguration, the kinematics of the lead screw and the velocity of the steering units moving towards or away from the central beam must obey the mechanical constrain that is seen in Figure 6. Based on the mechanical constrain, the velocity of steering unit motors and lead screw motor have to be always synchronized to maintain the Panthera aluminum frame where $s=\sqrt{L_{arm}^{2}-b^{2}}$. To achieve that, we differentiate the mechanical constraint to understand the relationship between the velocity of the carriage, $\dot{s}$, which is driven by the lead screw, and the velocity of the side beams moving towards or away from the central beam, which is driven by the steering units. A robust reconfiguration kinematics model is shown below.

#### 6.1.1. Robust Reconfiguration Kinematics

In our previous work [29], we developed the reconfiguration kinematics for three cases, which are, as follows:

- Case I: Reconfiguration in width where the left side beam remains static in the robot frame.
- Case II: Reconfiguration in width where the right beam remains static in the robot frame.
- Case III: Reconfiguration in width where the central beam remains static in the robot frame.

Based on these three cases, Panthera was not robust enough to take in continuous reconfiguration input based on the vision-based perception algorithm. It was only able to receive a command to perform either of the three cases.

We proposed a robust reconfiguration kinematics, where Panthera is able to reconfigure with respect to the central beam, where both side beams can be moving at a different speed. This enables the vision-based feedback control to pass on kinematics input for reconfiguration continuously and allowing Panthera to reconfigure beyond the three cases that were highlighted in our previous work. Figure 14 shows the reference frame used in the kinematics equations.

For reconfiguration with respect to the central beam, with both side beams moving into or out of the robot at different speed, the two side beams and the central beam are moving in the robot reference frame while compressing or expanding. $0<\delta_{1}=\delta_{4}<\pi /2$, while $-\pi /2<\delta_{3}=\delta_{2}<0$ and $\delta_{4}=\delta_{1}\neq -\delta_{3}=-\delta_{2}$. The velocity in the x and y component are as follows:

(17) $$v_{yi}=v_{i}sin(|\delta_{i}\left|\right)$$

(18) $$v_{xi}=v_{i}sin(\pi -|\delta_{i}\left|\right)\;for\;i=1,2,3,4$$

The speed at which the steering units move towards or away from the central beam, $v_{y}$, equals the rate of change of $b_{MR}$, ${\dot{b}}_{MR}$. The robust reconfiguration kinematics can have a moving centroid, where the central beam, and the speed of the centroid in the y direction in the Panthera reference frame, $v_{yc}$, is as follows:

(19) $$\begin{array}{cc} v_{yc} & =\frac{v_{y1}-v_{y2}}{2}\\ \; & =\frac{v_{y4}-v_{y3}}{2} \end{array}$$

The speed of the lead screw motor during reconfiguration with respect to the central beam is as follows:

(20) $$\begin{array}{cc} {\dot{b}}_{MR} & =\frac{v_{y1}+v_{y2}}{2}\\ \; & =\frac{v_{y4}+v_{y3}}{2} \end{array}$$

(21) $$\dot{s}=\frac{-{\dot{b}}_{MR}b_{MR}}{\sqrt{L_{arm}^{2}-b_{MR}^{2}}}$$

Based on the robust kinematics model, Case I, Case II, and Case III can be performed at specific kinematics parameters, as shown in the next few sections.

#### 6.1.2. Case I and Case II

During reconfiguration in Case I and Case II, the robot one of the side beams will not move in the robot reference frame, while the opposite side beam will move either away or towards the center of the robot, depending on compression or expansion mode. The steering units on the side beam, which does not move in the robot reference frame, will have zero steering angles, while the opposite side beam will have steering angles where the mechanical constrain is adhered to. Let $V_{j}$ be the speed of the $SU_{1}$ and $SU_{4}$ for Case I and $SU_{2}$ and $SU_{3}$ for Case I, while $V_{k}$ is the speed of the $SU_{3}$ and $SU_{3}$ for Case I and $SU_{1}$ and $SU_{4}$ for Case II.

(22) $$\begin{array}{cc} v_{yj} & =v_{j}sin(|\delta_{i}\left|\right)\\ \; & =\frac{v_{rj}+v_{lj}}{2}sin(|\delta_{j}\left|\right)\\ \; & =\frac{r\varphi_{rj}+r\varphi_{lj}}{2}sin(|\delta_{j}\left|\right)\\ \; & =2{\dot{b}}_{MR} \end{array}$$

(23) $$\begin{array}{cc} v_{xj} & =v_{j}cos(|\delta_{i}\left|\right)\\ \; & =\frac{v_{rj}+v_{lj}}{2}cos(|\delta_{j}\left|\right)\\ \; & =\frac{r\varphi_{rj}+\varphi_{lj}}{2}cos(|\delta_{j}\left|\right) \end{array}$$

(24) $$v_{xk}=v_{j}cos(|\delta_{i}\left|\right)=\frac{v_{rj}+v_{lj}}{2}cos(|\delta_{j}\left|\right)$$

(25) $$v_{yk}=0$$

For reconfiguration with left side beam that is not moving in the robot reference frame, ($SU_{1}$, $SU_{4}$) will have zero steering angle $\delta_{1}=\delta_{4}=0$, whereas ($SU_{2}$, $SU_{3}$) have $-\pi /2<\delta_{2}=\delta_{3}<0$. For reconfiguration with right side beam not moving in the robot reference frame, the steering unit heading angles are $0<\delta_{1}=\delta_{4}<\pi /2$ while $\delta_{2}=\delta_{3}=0$.

(26) $$\begin{array}{cc} {\dot{s}}_{Case-I} & ={\dot{s}}_{Case-II}\\ \; & =\frac{-{\dot{b}}_{MR}b_{MR}}{\sqrt{L_{arm}^{2}-b_{MR}^{2}}} \end{array}$$

#### 6.1.3. Case III

For reconfiguration with respect to the central beam, the robot central beam is not moving in the robot reference frame during its compression or expansion, where $0<\delta_{4}=\delta_{1}<\pi /2$ and $-\pi /2<\delta_{3}=\delta_{2}<0$ and $\delta_{4}=\delta_{1}=-\delta_{3}=-\delta_{2}$. The velocity in the x and y component are as follows:

(27) $$v_{yi}=v_{i}sin(|\delta_{i}\left|\right)$$

(28) $$v_{xi}=v_{i}sin(\pi -|\delta_{i}\left|\right)\;for\;i=1,2,3,4$$

The speed at which the steering units move towards or away from the central beam, $v_{y}$, equals the rate of change of $b_{MR}$, ${\dot{b}}_{MR}$.

(29) $$\begin{array}{cc} v_{yi} & =v_{i}sin(|\delta_{i}\left|\right)\\ \; & =\frac{v_{ri}+v_{li}}{2}sin(|\delta_{i}\left|\right)\\ \; & =\frac{r\varphi_{r}i+r\varphi_{li}}{2}sin(|\delta_{i}\left|\right)\\ \; & ={\dot{b}}_{MR} \end{array}$$

The speed of the lead screw motor during reconfiguration with respect to the central beam is as follows:

(30) $${\dot{s}}_{Case-III}=\frac{-{\dot{b}}_{MR}b_{MR}}{\sqrt{L_{arm}^{2}-b_{MR}^{2}}}$$

**Figure 16:**
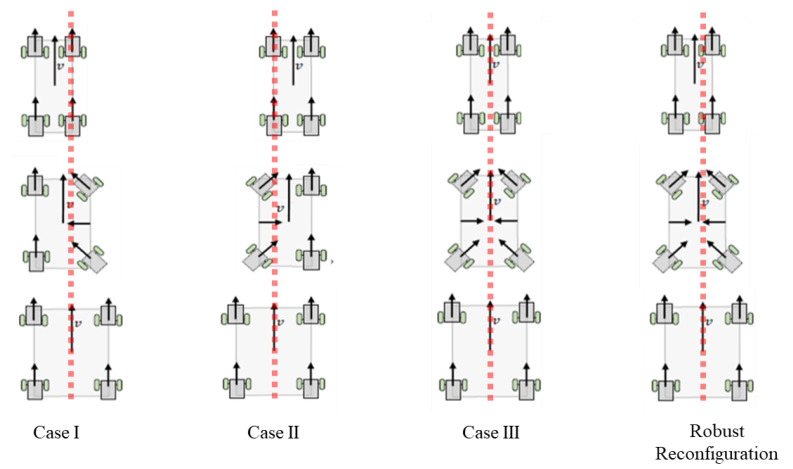
Panthera gaits for reconfiguration during locomotion.

Figure 16 shows a visual example of Case I, Case II, and Case III and the robust kinematic model. Panthera is also capable of performing reconfiguration via expansion.

Using the robust reconfiguration kinematics equations presented above, Panthera will be able to reconfigure Case I, Case II, and Case III, as highlighted in our previous work. In addition, it is able to perform beyond our previous work, as Panthera will now be able to reconfigure with different steering inputs for both the left and right side beams. This allows for the vision-based algorithm to pass in our proposed beta left and beta right inputs for kinematics control. The equation is applicable for both compression or expansion in width. Panthera is also capable of performing static reconfiguration, where $v_{xi}=0$. In the next section, the experimental results for the Panthera perception feedback algorithm derive two parameters, $\beta_{l}$ and $\beta_{r}$. The parameters are published in the processing node and subscribed by the locomotion and reconfiguration node for the reconfiguration and control of Panthera. Using the two parameters, Panthera will be able to perform locomotion and reconfiguration with the kinematics equations that are described in this section.

## 7. Experimental Results

**Figure 17:**
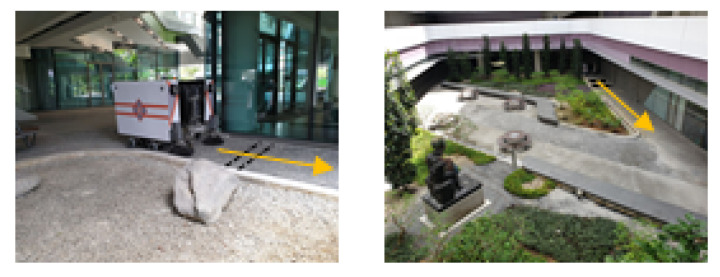
Panthera platform parallel to pavement.

**Figure 18:**
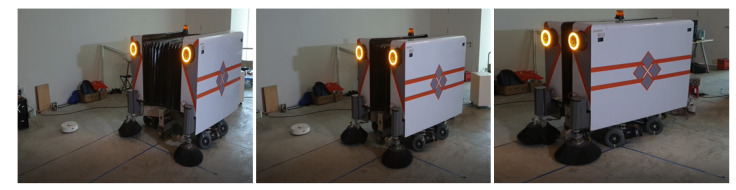
Reconfiguration during locomotion where central beam moves along the y direction in the robot reference frame.

For a good evaluation of Panthera’s capability to avoid pedestrians while adapting to surroundings through reconfiguration in width, the experiments are conducted on a straight pavement with a pedestrian walking towards and away from Panthera. Panthera was set as in Figure 17. A straight pavement is chosen to demonstrate the reconfiguration based on pedestrians more clearly. Only humans were assumed as obstacles in the pavement in all of the experiments, and the robust reconfiguration kinematics of the reconfiguration was performed during locomotion with the input beta left and beta right values from the vision system. A total of six experiments are conducted, of which three experiments are conducted using the Central Processing Unit (CPU) and, similarly, three experiments are conducted using the Graphics Processing Unit (GPU). In the first experiment, a pedestrian will be walking towards Panthera while Panthera is moving at a constant speed. The first experiment is repeated using a CPU and a GPU. In the second experiment, a pedestrian will be walking away from Panthera while Panthera is moving at a constant speed. The second experiment is repeated while using a CPU and a GPU. In the third and last experiment, a pedestrian starts walking towards Panthera and then moving away from Panthera. The third experiment is also repeated using a CPU and a GPU. In all experiments, the depth value of pedestrian, estimated depth value of pedestrian, estimated relative velocity of pedestrian, and the beta left and beta right values are provided. The beta left and beta right values will serve as input parameters for reconfiguration during locomotion, as shown in Figure 18.

**Figure 19:**
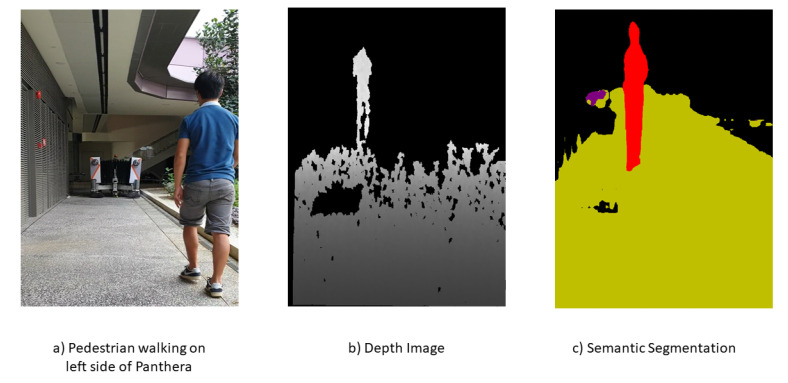
Pedestrian on the left of Panthera.

In all experiments, semantic segmentation is applied to the stream of RGB images from the RealSense D435 camera sensor. The semantic segmentation Mobilenet Deeplab model is used for both CPU and GPU experiments, as seen in Figure 19c. More information of the semantic segmentation model can be found in Appendix B. The semantic segmentation model is trained on the CoCo dataset [48]. The CoCo dataset is a repository of large-scale object detection, segmentation, and captioning datasets. The average image pixels coordinates of pavement edges are extracted from two sections of images, and pixel coordinates of the pedestrian were also extracted after using semantic segmentation for classification. Homogeneous Equation (4) are used for converting pixel to real-world coordinates.

**Figure 20:**
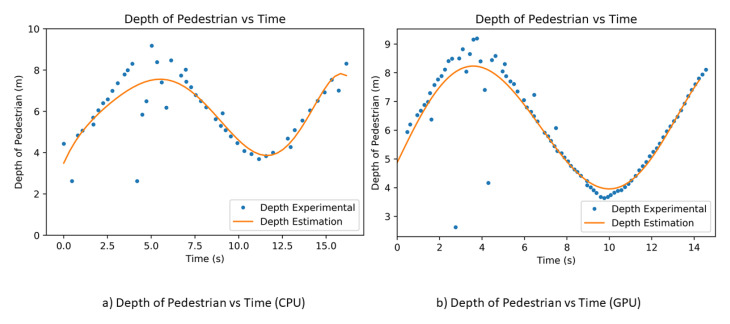
Pedestrian depth against time.

In order to determine the performance of estimating the pedestrian depth and relative velocity, we performed an experiment where the pedestrian moves away and towards Panthera. We used the polyfit to estimate the pedestrian’s actual depth value in order to reduce the noise of the pedestrian’s depth value. There are many other methods available for predicting trajectory, speed estimation, and prediction, especially in the automobile industry, such as in [49]. In our paper, the gradient of the polyfit was used to estimate the velocity of the pedestrian. The experiment is also conducted with both CPU and GPU, and the results are seen in Figure 20. From the CPU and GPU performance, the GPU has a better depth estimation, as it has more data points for a more accurate result. A more accurate depth estimation will result in a more accurate depth estimation and it will affect the beta left and beta right vision output parameters for the reconfiguration during locomotion. GPU performs at a frame rate of 10 fps (frames per second), while CPU performs at a frame rate of 3 fps. It is noted that the range of the RealSense D435 camera is up to about 10 m and, when the pedestrian is more than 10 m away, the depth value of the pedestrian is not accurate.

### 7.1. First Experiment

**Figure 21:**
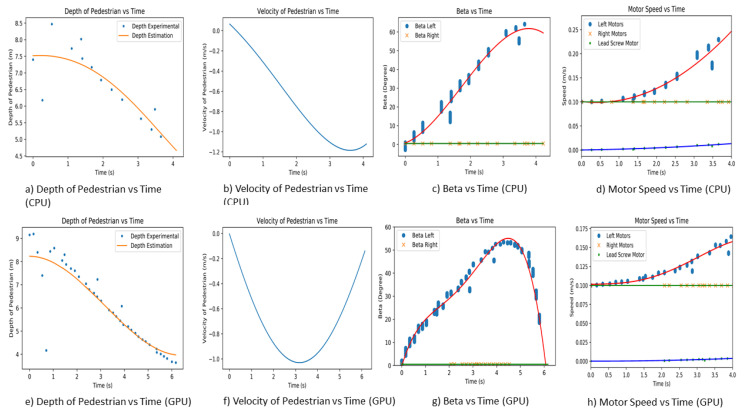
Experimental results for first experiment.

The first experiment is performed on a straight pavement, where Panthera is moving at a constant speed of 0.1 m/s. The pedestrian will commence walking when the experiment starts. The experiment is repeated for both CPU and GPU. The pedestrian is walking parallel to the pavement and on the left side of Panthera, as seen in Figure 19. The actual depth and depth estimation of the pedestrian estimated relative velocity and beta left and beta right is plotted against time for both CPU and GPU, as seen in Figure 21. At the start of the experiment, the pedestrian is position at 9 m away from Panthera. As the pedestrian walks towards Panthera, the depth value and depth estimated value of the pedestrian decreases. As the CPU has a frame rate of 3 fps while the GPU has a frame rate of 10 fps, the GPU can produce higher-resolution depth data, which results in a better estimation of the pedestrian velocity. Using the estimated velocity, the pixel coordinates of the pedestrians, and pavement edges, the beta left and beta right values are derived. In both CPU and GPU experiments, as the pedestrian is walking on the left side of Panthera, beta right remains small and close to 0. On the other hand, beta left increases as the pedestrian walks towards Panthera. Beta left generally increases proportionally to the estimated relative velocity of the pedestrian. The noise in the beta values is due to the imperfections of the semantic segmentation and the conversion of pixel coordinates to world coordinates.

The left and beta right values are passed to the Panthera locomotion and reconfiguration node. The proportional Integral Differential (PID) controller is for controlling the respective motors that are based on the left and beta right values. The PID controller is used for handling the motor speed and steering angle of the steering unit. Here, the control logic takes care of minimizing error and auto-tuning the PID values for better performance of Panthera. Control logic provides the left motors ($SU_{1}$, $SU_{4}$) speed, right motors ($SU_{2}$, $SU_{3}$) speed and lead screw speed, as seen in Figure 21d,h. As the left beta values increase, the left motors ($SU_{1}$, $SU_{4}$) increase to compensate for the speed that is responsible for the reconfiguration component. As the right beta values are relatively low, the speed of the right motors ($SU_{2}$, $SU_{3}$) remains close to the robot speed. Lead screw motor speed is also seen to be increasing as the robot reconfigures.

### 7.2. Second Experiment

**Figure 22:**
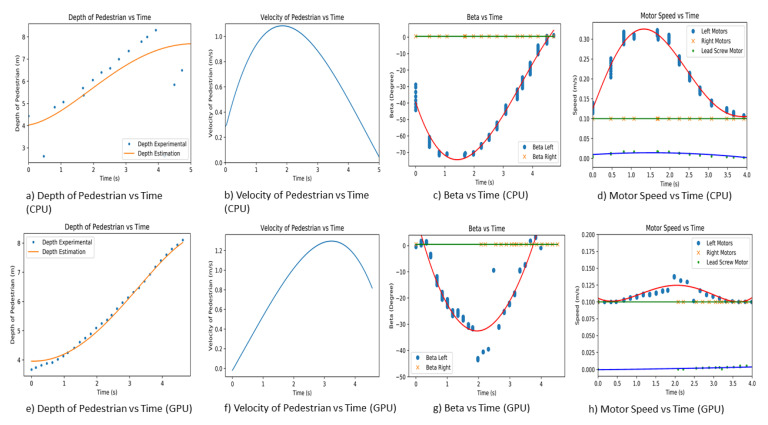
The experimental results for second experiment.

The second experiment is performed on a straight pavement, where Panthera is moving at a constant speed of 0.1 m/s. The pedestrian will commence walking away from Panthera when the experiment starts. The experiment is repeated for both CPU and GPU. The pedestrian is walking parallel to the pavement and on the left side of Panthera. The actual depth and depth estimation of the pedestrian estimated relative velocity and beta left and beta right is plotted against time for both CPU and GPU, as seen in Figure 22. At the start of the experiment, the pedestrian is position at 4 m away from Panthera. As the pedestrian walks away from Panthera, the depth value and depth estimated value of the pedestrian increases. As the CPU has a frame rate of 3 fps while the GPU has a frame rate of 10 fps, the GPU is able to produce higher-resolution depth data, which results in a better estimation of the pedestrian velocity. Using the estimated velocity, pixel coordinates of the pedestrians, and pavement edges, the beta left, and beta right values are derived. In both CPU and GPU experiments, as the pedestrian is walking on the left side of Panthera, beta right remains small and close to 0. On the other hand, beta left increases in magnitude in the negative direction as the pedestrian walks towards Panthera. Beta left generally increases proportionally to the estimated relative velocity of the pedestrian. Noise in the beta values is due to the imperfections of the semantic segmentation and the conversion of pixel coordinates to world coordinates.

The left and beta right values are passed to the Panthera locomotion and reconfiguration node. The proportional Integral Differential (PID) controller is for controlling the respective motors based on the left and beta right values. The PID controller is used for handling the motor speed and steering angle of the steering unit. Here, the control logic takes care of minimizing the error and auto-tuning the PID values for better performance of Panthera. Control logic provides the left motor ($SU_{1}$, $SU_{4}$) speed, right motor ($SU_{2}$, $SU_{3}$) speed, and lead screw speed, as seen in Figure 22d,h. As the left beta values increase in magnitude, the speed of the left motors ($SU_{1}$, $SU_{4}$) increases to compensate for the speed that is responsible for the reconfiguration component. Because the right beta values are relatively low, the speed of the right motors ($SU_{2}$, $SU_{3}$) remains close to the robot speed. Lead screw motor speed is also seen to be increasing as the robot reconfigures in width.

### 7.3. Third Experiment

The third experiment is performed on a straight pavement, where Panthera is moving at a constant speed of 0.5 m/s. At the start of the experiment, the pedestrian runs towards Panthera, makes a U-turn, and then walks away from Panthera. Similarly, the pedestrian is on the left side of Panthera, and the experiment is repeated for both CPU and GPU. Because the CPU has a frame rate of 3 fps while the GPU has a frame rate of 10 fps, the GPU is able to produce higher-resolution depth data, resulting in a better estimation of the pedestrian velocity. The actual depth and depth estimation of the pedestrian estimated relative velocity and beta left and beta right is plotted against time for both CPU and GPU.

**Figure 23:**
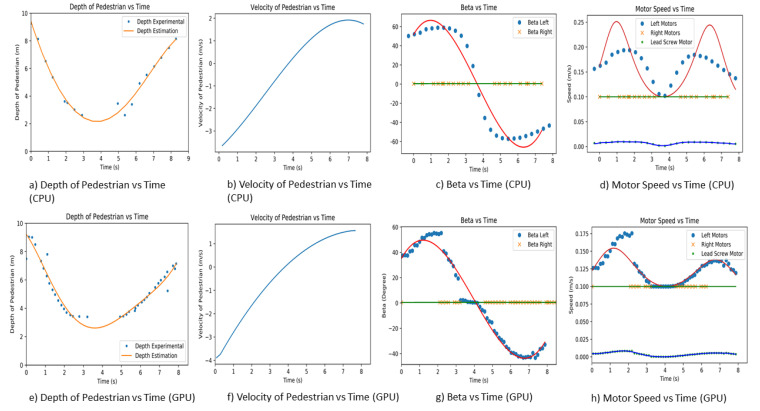
Experimental results for third experiment.

At the start of the experiment, the pedestrian is in position at 10 m away from Panthera. From the initial position, the pedestrian will run towards Panthera and, when the pedestrian is close to Panthera, the pedestrian will turn around and walk away from Panthera. At the start, when the pedestrian runs towards Panthera, the depth value of the pedestrian drops. As the pedestrian change direction and walks away from Panthera, the depth value increases correspondingly. This is reflected in Figure 23, where the depth value and estimated depth value for both CPU and GPU decreases and then increases. This is also reflected in the velocity where the pedestrian’s velocity is initially negative and slowly becomes positive. When the pedestrian is running towards Panthera, the magnitude of velocity is higher than when the pedestrian is walking away from Panthera. Using the estimated velocity, pixel coordinates of the pedestrians, and pavement edges, the beta left and beta right values are derived. In both CPU and GPU experiments, as the pedestrian is walking on the left side of Panthera, beta right remains small and close to 0. On the other hand, beta left increases in magnitude in the negative direction as the pedestrian walks towards Panthera. Beta left generally increases proportionally to the estimated relative velocity of the pedestrian. The noise in the beta values is due to the imperfections of the semantic segmentation and the conversion of pixel coordinates to world coordinates.

The beta left and beta right values will be passed on to the Panthera locomotion and reconfiguration node. Panthera will use these values as input to the respective motors, and it is controlled by a proportional integral differential (PID) control. With the PID control of Panthera of the motors speed and steering angle of the steering units, the control logic minimizes the error quotient and auto-tunes the PID values for the better drivability of the Panthera, providing the desired left motor ($SU_{1}$, $SU_{4}$) speed, right motor ($SU_{2}$, $SU_{3}$) speed, and lead screw speed, as seen in Figure 22d,h which are required to perform the reconfiguration during locomotion, as discussed in [29]. As the pedestrian moves towards and then away from the robot’s left side, the left motors ($SU_{1}$, $SU_{4}$) have to increase their speed to compensate for the compression and expansion motion. Because the right beta values are relatively low, the speed of the right motors ($SU_{2}$, $SU_{3}$) remains close to the robot speed. The lead screw motor speed is also seen to be increasing as the robot reconfigures in width.

The three experiments demonstrate that the proposed vision algorithm enables Panthera to understand pedestrian estimated position and velocity and to perform vision-based kinematics control using the robust reconfiguration kinematics. It provides beta left and beta right, which are key parameters that will enable the robot to change the left motors ($SU_{1}$, $SU_{4}$) steering angle and right motors ($SU_{2}$, $SU_{3}$) steering angles. Based on these steering angles, the robot can calculate its motor speed in order to perform locomotion and reconfiguration while it is still moving. These key parameters enable Panthera to be adapt its reconfiguration state based on pedestrians and non-symmetrical pavement shape, which was not possible in our previous work. With our proposed algorithm, Panthera will be aware of pedestrians in the dynamically changing pavement environment.

## 8. Conclusions and Future Work

In our previous work, Panthera is only capable of adapting to width-changing symmetrical pavements through Case III reconfiguration kinematics. In this research, we studied how Panthera can understand the surroundings that it is in and avoid a pedestrian that is in its way. The processing node’s ability to understand its surroundings and pass on parameters to the locomotion and reconfiguration node for control. The model detects the pavement width and pedestrians while using the mask-based deep convolutional neural networks (DCNN) and depth image. This allows the robot to gain information regarding the pavement and the position of the pedestrian. This enables the robot to change the width according to the output parameters of beta left and beta right to avoid pedestrians during its cleaning operations. We have also developed a more robust reconfiguration kinematic model that enables Panthera to take in the beta left and beta right parameters for reconfiguration to avoid pedestrians and adapt to non-symmetrical pavement shapes.

Future work will involve enabling Panthera to avoid additional dynamic and static obstacles, such as animals and potholes, through different classes in the semantic segmentation. We will also work on using a combination of RGB-D camera and lidar for better position and speed estimation of dynamic and static obstacles. The vision feedback-based pedestrian avoidance and kinematics models are reported in the literature. Hence, it can be added as part of future work.

## Figures and Tables

**Table 1:** Current pavement sweeping machines.

Specification	MN-E800W	QS3008	CN 201
Power supply	48 V	48 V	Diesel
Holonomic	Non-holonomic	Non-holonomic	Non-holonomic
Length	2150 mm	2560 mm	4270 mm
Width	1900 mm	1110 mm	1315 mm
Height	2040 mm	1880 mm	1985 mm
Payload	180 kg	80 kg	1700 kg

## Data Availability

Not applicable.

## Appendix A. Terminologies and Definitions

**Symbol**	**Definition**
$p_{leadscrew}$	Pitch of lead screw
$Z_{SU_{i}}$	Steering axis of the robot: Directed out
$X_{SU_{i}}$	Steering axis of the robot: Directed upwards
$Y_{SU_{i}}$	Steering axis of the robot: Directed left
$w_{i}$	Pavement width in section i
*w*	Pavement width
$d_{i}$	Depth value of section i
$d_{k}$	Depth value per pixel
$\Omega_{i}$	Number of pixels in section *i*
$f_{x}$	Focal length of camera in pixel x axis
$f_{y}$	Focal length of camera in pixel y axis
$c_{x}$	Optical center in pixel x axis
$c_{y}$	Optical center in pixel y axis
*P*	Camera matrix
*R*	Rotational Vector
*T*	Translation Vector
*E*	Camera extrinsic matrix
*W*	World coordinate
*p*	Pixel coordinate
$c_{x}$	Optical center in pixel x axis
$c_{y}$	Optical center in pixel y axis
$x_{li}$	Average of minimum x coordinate in section i
$x_{ri}$	Average of maximum x coordinate in section i
$y_{li}$	Average of minimum x coordinate in section i
$y_{ri}$	Average of maximum x coordinate in section i
$d_{li}$	Depth of minimum x coordinate in section i
$d_{ri}$	Depth of maximum x coordinate in section i
$x_{cp}$	Center x pixel coordinate of the pedestrian
$w_{p}$	Center x world coordinate of the pedestrian
$Min(w_{p})$	Minimum x coordinate of the pedestrian
$Max(w_{p})$	Maximum x coordinate of the pedestrian
Δw	Width in section 1 minus width in section 2
Δd	Depth in section 1 minus depth in section 2
$d_{li}$	Maximum Depth x coordinate in section i
$d_{ri}$	Maximum Depth x coordinate in section i
β	Derive required steering angle δ
$\beta_{l}$	Derive required steering angle for $SU_{1},SUI_{4}$
$\beta_{r}$	Derive required steering angle for $SU_{2},SUI_{3}$
*v*	Velocity of robot
$v_{x}$	X component of velocity robot
$v_{y}$	Y component of velocity robot
$v_{i}$	Velocity of steering unit i
$v_{xi}$	x component velocity of steering unit i
$v_{yi}$	y component velocity of steering unit i
$x_{SU_{i}}^{R}$	X coordinates of ith steering units in robot frame
$y_{SU_{i}}^{R}$	Y coordinates of ith steering units in robot frame
$\delta_{i}$	Steering angle of $SU_{i}$: Angle between $v_{i}$ and $X_{S}Ui$
*r*	Radius of wheel = 108 mm
*b*	Distance between center and side beam
$\dot{b}$	Distance change Rate center and side beam
$\dot{s}$	The velocity of the carriage
${\dot{s}}_{Case-I}$	Velocity of carriage for Case-I
${\dot{s}}_{Case-II}$	Velocity of carriage for Case-II
${\dot{s}}_{Case-III}$	Velocity of carriage for Case-III
$b_{MR}$	Distance between center and the side beam
${\dot{b}}_{MR}$	Change rate between center and side beam
$L_{arm}$	Length of linkage bar
ω	Angular velocity of robot
$V_{j}$	Speeds of steering units
$v_{rj}$	Right wheel speeds of steering units
$v_{lj}$	Left wheel speeds of steering units
${\dot{\varphi }}_{rj}$	Right wheel angular velocity of steering units
${\dot{\varphi }}_{lj}$	Left wheel angular velocity of steering units
$V_{k}$	Reconfiguration Steering units Speed
$v_{xk}$	X component speeds of steering units
$v_{yk}$	Y component speeds of steering units
$v_{rk}$	Right wheel speeds of steering units
$v_{lk}$	Left wheel speeds of steering units
${\dot{\varphi }}_{rk}$	Right wheel angular velocity of steering
${\dot{\varphi }}_{lk}$	Left wheel angular velocity of steering

## Appendix B. Mask Based DCNN Network

In Deeplabv3, Atrous Spatial Pyramid Pooling (ASPP) is employed with encoder structure for obtained the rich semantic information. Detailed object boundaries are recovered by the decoder module. The encoder features are first bilinearly up sampled by a factor of 4 and then concatenated with the corresponding low-level features. There is 1 × 1 convolution on the low-level features before concatenation to reduce the number of channels, since the corresponding low-level features usually contain a large number of channels (e.g., 256 or 512) which may outweigh the importance of the rich encoder features. After the concatenation, a few 3 × 3 convolutions is used to refine the features followed by another simple bilinear up sampling by a factor of 4. MobileNetv2 Deeplabv3 is a version of DeepLab [42], a state-of-the-art semantic segmentation model, which employs MobileNet v2 as its backbone for light weight processing.
